# Self-care support of diet and the gut in the routine care of school-age children with long-term conditions: An integrative review

**DOI:** 10.1177/13674935211029124

**Published:** 2021-06-30

**Authors:** Laurie Cave, Linda J Milnes, Gretl A McHugh

**Affiliations:** 1 School of Healthcare, 4468 University of Leeds, Leeds, UK; 2 Children’s Nutrition and Dietetics, 4472 Leeds Teaching Hospitals NHS Trust, Leeds, UK

**Keywords:** Child, diet, gastrointestinal, self-care, systematic review

## Abstract

There is policy impetus for provision of self-care support (SCS) for children with long-term conditions (LTCs). However, it is not clear what SCS should consist of and how it can be delivered in routine care. This review aimed to synthesise the literature, specifically on SCS of diet and the gut as these components are essential for optimal growth and development and enhanced quality of life. Using an integrative review methodology, studies conducted between January 1990 and July 2020 were systematically identified and methodological quality assessed using the Mixed Methods Appraisal Tool. Twenty-five studies were included. SCS of diet and the gut consisted of support in developing and applying specific knowledge and skills and practical help with incorporating the demands of self-care into everyday life. Key requisites for models of SCS in the context of delivery and uptake in routine care were starting early, keeping it going, being flexible and choosing appropriate outcomes. This review contributes new understanding on the provision of SCS of diet and the gut for school-age children with LTCs, including identification of gaps in the literature and further research needs.

## Introduction

Enabling school-age children with long-term conditions (LTCs) to take an active role in their care is essential if the development of life-long self-care skills, attitudes and behaviours is to become a normal, expected aspect of routine care (Fung, 2020; Taylor et al., 2014).

Self-care may be defined as the broad range of activities carried out to *live well with a LTC* (Kirk et al., 2010). The term ‘self-care’ is often used interchangeably with ‘self-management’; however, self-management more narrowly relates to managing *the LTC well* (Pelicand et al., 2013). Self-care incorporates self-management and also health promotion (Bee et al., 2018). This broad perspective is particularly appropriate in the care of children with LTCs, for whom developmental, psychosocial and healthcare needs are closely intertwined (Aujoulat et al., 2006) and where anticipatory guidance may pre-empt some of the challenges faced.

Whilst the burden of performing daily self-care rests with patients and their families, healthcare professionals (HCPs) can help to enable self-care. There is policy impetus to support the self-care of children with LTCs in the United Kingdom (Department of Health and Department for Education and Skills, 2004; NHS England, 2014, 2019). However, it is not clear what self-care support (SCS) should consist of and how it can be delivered as part of routine care. This uncertainty is important to address as laying a solid foundation for self-care in childhood may better prepare children for living well over their life course, to navigate challenges during adolescence (Christian and D’Auria, 2006) and to improve outcomes in adulthood (Culhane et al., 2013).

In LTCs with a diet and/or gastrointestinal (GI)-related component of care, a specific focus on SCS of these components is warranted. This is because self-care of diet and/or the gut is essential for controlling symptoms and optimising children’s growth and development, which, in turn, may enhance quality of life (Shoff et al., 2013; Spinks and Guest, 2017; Rocha et al., 2013; Wagner et al., 2008). Although diet and the gut are central to care in cystic fibrosis (CF) (Turck et al., 2016), studies on SCS in CF are limited. This review therefore sought to learn from a broad range of other childhood-onset LTCs with a diet and/or GI component, such as type 1 diabetes (T1DM), as a first step in developing a model for SCS of diet and the gut in CF.

Two systematic reviews on SCS for children with LTCs have been conducted (Bee et al., 2018; Kirk et al., 2013). Both provide some evidence to inform the development of a model for SCS. For example, SCS interventions that directly targeted children were effective, particularly in improving psychosocial well-being (Kirk et al., 2013). However, in both systematic reviews, the included studies predominantly focused on children with asthma and therefore did not consider self-care of diet and/or the gut. In addition, the findings of both are limited with respect to informing how SCS can be delivered as part of routine care. Further evidence synthesis is therefore required, of both qualitative and quantitative studies, to move beyond examining effectiveness and obtain a comprehensive understanding of context (Pawson et al., 2005).

For the current review, an integrative review methodology was chosen to systematically capture breadth and depth through integrating findings from qualitative, quantitative and mixed methods studies in a single synthesis (Leeman et al., 2015). The aim of this review was to identify, critically appraise and synthesise evidence from primary studies on SCS of diet and the gut in school-age children with LTCs, to obtain a comprehensive understanding of what is already known, what the gaps are and what new knowledge is needed.

Two review questions were addressed:

• What is SCS of diet and the gut for school-age children with LTCs?
• What models of SCS have worked, when and how, in the routine care of school-age children with LTCs (including enablers for and barriers to, delivery and uptake)?

## Methods

This review was conducted in accordance with the methodological guidelines of Whittemore and Knafl (2005). A protocol was developed and registered with PROSPERO (CRD42019144941) (Cave et al., 2019). The reporting of the review followed the Preferred Reporting Items for Systematic review and Meta-Analyses (PRISMA) guidance (Moher et al., 2009).

### Inclusion criteria

This review considered studies (i) that included children of school-age (4–16 years) with any physical LTC with a diet and/or GI-related component of care or the following exemplar LTCs: CF, T1DM, coeliac disease, phenylketonuria or inflammatory bowel disease; (ii) in which children were actively involved in self-care of diet and/or the gut having received some type of SCS and/or (iii) investigated enablers for and barriers to, delivery and uptake of SCS. All types of studies were considered: qualitative, quantitative and mixed methods. The inclusion and exclusion criteria for the review are detailed in Supplementary Material 1.

### Search strategy

The search strategy aimed to find both published literature and grey literature. A comprehensive search of electronic databases was conducted, including CINAHL, Medline, Embase, PsycINFO, Scopus, Web of Science and the Cochrane Library. Additional information sources for grey literature and unpublished studies included OpenGrey, the ISRCTN registry and ClinicalTrials.gov.

The search dates in each database were from January 1990 to July 2020. This date range reflects the development of policy and research in self-care/self-management of LTCs since the 1990s. The search strategy was modified for each database*,* included use of database-specific subject headings, free-text terms and variations relating to diet/gut self-care, children and LTCs. An example of the search strategy is included in Supplementary Material 2.

Additional search strategies included citation searching, targeted author searches and hand searching reference lists of included studies, review articles and key journals. Searches were limited to studies published in English.

### Study selection

Following the search, all potentially eligible studies were collated and duplicates removed. A two-stage screening process was adopted. Stage one involved the screening of titles and abstracts. Studies that met the inclusion criteria were taken forward to stage two which involved full-text screening of the studies against the inclusion criteria. Where required, companion papers were sought or authors emailed to request missing or additional information (Hong et al., 2018). Both stages were led by LC, independently checked by LM and GM and consensus reached by discussion.

### Data extraction

Data were extracted from the eligible studies using a pre-piloted data extraction table. The data extracted included research aim, study design and methods, participants, intervention/exposure to SCS, setting, key findings and supporting data for quality appraisal. Data were extracted by LC and 10% of the full extraction checked for accuracy and completeness by LM and GM. Any discrepancies that arose between the reviewers were resolved through discussion.

### Quality appraisal

The methodological quality of included studies was critically appraised using the Mixed Methods Appraisal Tool (MMAT) (version 2018) (Hong et al., 2018). MMAT was chosen as it is a validated tool, has comprehensive guidelines and allowed concomitant appraisal of core methodological criteria for the included study designs. Quality appraisal was conducted by LC and 10% of the full assessment independently checked by LM and GM. Any discrepancies that arose between the reviewers were resolved through discussion.

### Data analysis and synthesis

Due to the heterogeneity across included studies, studies could not be combined statistically, and a narrative analysis and synthesis were therefore conducted. Data extracted from quantitative studies were converted into textual descriptions to facilitate integration with data from qualitative studies. Assembled data were then systematically coded, organised into categories and iteratively compared across the studies to begin identifying patterns, variations and relationships (Whittemore and Knafl, 2005). LC conducted the analysis and developed a set of integrated findings in the form of themes. Themes were verified following discussion with all authors and presented as a narrative summary.

## Results

### Study inclusion

**Figure 1.**
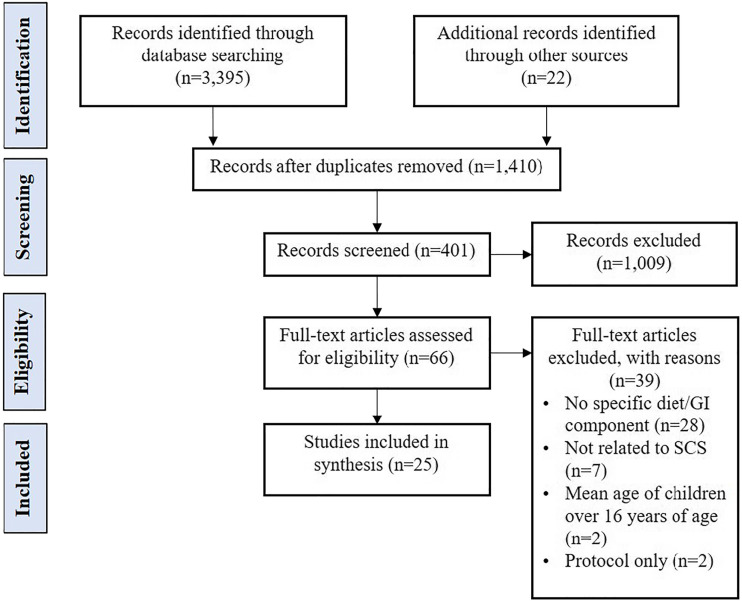
Selection process: PRISMA flow diagram (Moher et al., 2009).

The search strategy identified 3417 records, summarised using a PRISMA flow diagram (Figure 1) (Moher et al., 2009). A total of 27 articles reporting on 25 studies met the review inclusion criteria.

### Description of the included studies

Characteristics of the included studies are summarised in Supplementary Material 3. Overall, studies included children and adolescents aged 2–19 years, predominantly with T1DM (*n* = 12, 48%) and CF (*n* = 9, 36%), followed by phenylketonuria (*n* = 2, 8%), coeliac disease (*n* = 1, 4%) and concurrent coeliac disease and T1DM (*n* = 1, 4%). No studies that included children and adolescents with inflammatory bowel disease met the inclusion criteria. Of the 25 studies, 18 were quantitative, five mixed methods and two qualitative designs; 12 were conducted in Europe, 11 in North America, with the remainder conducted in Australia and Brazil.

The majority of studies (*n* = 19, 76%) provided models of SCS, whilst other studies (*n* = 6, 24%) informed the context of SCS. Several studies had limited detail about the diet/GI-related component of care (Cooper et al., 2018; Cottrell et al., 1996; Fiallo-Scharer et al., 2019; Nabors et al., 2014), the dietary self-care programme (Austin et al., 2011, 2013) or routine support (Rankin et al., 2018a). However, they were included, as they were potentially relevant in answering the review questions.

### Quality appraisal

Only one study had insufficient information for appraisal using MMAT (Bell, 2004). Overall, whilst the majority of studies appraised had some limitations, they generally rated fair to good. No studies were excluded based on these findings though this was taken into consideration in the synthesis, in judging the strength of evidence for developing themes. MMAT findings are presented in Supplementary Material 4.

### Findings of the review

Six themes were identified through synthesis of the included studies. Two themes related to the first review question: (i) support in developing and applying specific knowledge and skills and (ii) practical help with incorporating the demands of self-care into everyday life. Four themes related to the second review question: (i) starting early, (ii) keeping it going, (iii) being flexible and (iv) choosing appropriate outcomes. Themes are described in the following narrative.

### First review question: What is SCS of diet and the gut for school-age children with LTCs?

#### Support in developing and applying specific knowledge and skills

There was consistency across the studies in supporting children and adolescents’ capability to self-care, beginning with development of essential, through to more advanced, knowledge and skills, with repeated opportunities to practice and develop confidence in their application.

A common starting point for school-age children in many of the studies was knowing how to identify which foods contained fat, carbohydrate (CHO), protein or gluten, as this set the foundation for selecting foods to eat, or restrict or avoid completely, as appropriate to the LTC (Boon et al., 2020; Christie et al., 2016; Fishman et al., 2018; Singh et al., 2000; Sparapani et al., 2017). Further knowledge was required to estimate how much fat, CHO, phenylalanine or protein was contained in foods (Coates et al., 2013; Witalis et al., 2017), possibly through developing the skill of reading food labels (Bell, 2004; Culhane, 2013; Rankin et al., 2018a). However, as this skill also relied on knowing how to estimate portion sizes, Spiegel et al. (2012) encouraged dietitians to support children and adolescents to repeatedly practice estimating portion sizes using real food and food models, alongside measuring actual portions. An alternative approach, in which adolescents with T1DM took photos of their own foods using an app (Frøisland and Årsand, 2015), had the advantage of facilitating estimation of both CHO content and portion sizes, and with further development, the app may be a useful tool for SCS.

More advanced knowledge was required to begin making sense of the complex relationships between CHOs, blood glucose and insulin (Coates et al., 2013; Christie et al., 2016; Frøisland and Årsand, 2015); fats, absorption and enzymes (pancreatic enzyme replacement therapy (PERT)) (Boon et al., 2020) and food containing gluten and absorption (Connan et al., 2019). In several studies, promoting a visual understanding of these relationships through use of an app (Frøisland and Årsand, 2015), an interactive e-learning module (Connan et al., 2019) or a video game (Sparapani et al., 2017), helped children and adolescents to make sense of what was happening inside their bodies as a result of coeliac disease and T1DM.

In many of the studies, the advanced skill of self-monitoring enabled recognition and management of GI symptoms (Bell, 2004; Cottrell et al., 1996; Culhane, 2013; Stapleton, 2001), hypo and hyperglycaemia (Nabors et al., 2014) and also tracking adherence to daily goals (Stapleton, 2001). Some studies combined building knowledge on how PERT or insulin works, with skills on administering and understanding what happens if too little or too much is taken (Bell, 2004; Davis et al., 2004). Further to this, studies focused on the advanced skill of titrating the dose of PERT to fat intake (Bell, 2004; Owen et al., 2013; Revert et al., 2018) and the dose of insulin to CHO (Christie et al., 2016; Coates et al., 2013; Price et al., 2016). Only one study (Rankin et al., 2018a), together with a companion study to Coates et al. (2013) (Chaney et al., 2010), highlighted poor mathematical comprehension as a barrier to performing this complex self-care task. Children adopted strategies to limit the need for complex maths skills such as choosing foods with CHO values they could remember or using mobile phones to contact their parents about CHO contents (thus remaining reliant on their parents) (Rankin et al., 2018a). In the trial of an app for CF, the enzyme dose calculation was the most used function by both children and their parents (Boon et al., 2020). This highlights numeracy as a vital core skill for self-care of diet and the gut in T1DM and CF.

#### Practical help with incorporating the demands of self-care into everyday life

Across many of the studies, SCS consisted of practical help for children and adolescents to have sustained opportunities and motivation to perform daily self-care.

In several studies, performing daily self-care relied on the creation of supportive physical environments, in which there was availability of planned foods for children with CF (Stapleton, 2001) and low protein foods for adolescents with phenylketonuria (Singh et al., 2000).

Children and adolescents’ ability to perform self-care also varied with the support received from HCPs, parents and friends (Austin et al., 2013; Boon et al., 2020; Frøisland and Årsand, 2015; Kyngäs et al., 1998; Rankin et al., 2018b; Revert et al., 2018; Singh et al., 2000; Spiegel et al., 2012; Stapleton, 2001; Witalis et al., 2017). Adolescents who felt their HCPs understood their dietary self-care challenges, accepted them as they were and provided them with choices were more motivated toward dietary self-care (Austin et al., 2013). Equally, children and adolescents valued HCPs who gave tailored advice and timely feedback (Boon et al., 2020; Frøisland and Årsand, 2015), particularly where this was simple practical advice relevant to their immediate situation and they could take action (Frøisland and Årsand, 2015). Kyngäs et al. (1998) highlighted the need for HCPs to ensure consultations are not dominated by disease-monitoring activities such as blood tests, to permit time for discussion of perceived barriers to self-care. In other studies, this extended to discussing other factors that may affect motivation to self-care, such as attitudes and beliefs in adolescents with phenylketonuria (Singh et al., 2000), goal setting in children with T1DM (Nabors et al., 2014) and emotions around food intake in children with T1DM (Sparapani et al., 2017).

Across several studies, HCPs provided practical support to parents in positively accepting their child’s growing independence (Witalis et al., 2017). They facilitated a balance of parents not exerting too much control (Austin et al., 2011; Kyngäs et al., 1998) or having too little involvement, as adolescents with T1DM who collaborated more with their parents had better metabolic control (Spiegel et al., 2012) and the value of an app to adolescents with T1DM was greater with parental support (Cooper et al., 2018). In one study, Rankin et al. (2018b) suggested HCPs could assist small friendship groups, to enable close friends of children with T1DM to provide support at school in the form of monitoring and prompting self-care tasks; furthermore, they suggested that raising awareness among school peers may help to normalise performance of self-care throughout the school day.

## Second review question: What models of SCS have worked, when and how, in the routine care of school-age children with LTCs (including enablers for and barriers to, delivery and uptake)

Four themes related to the second review question: (i) starting early, (ii) keeping it going, (iii) being flexible and (iv) choosing appropriate outcomes.

### Starting early

Across the included studies, models of SCS were more successful when started early in the disease course or in early childhood (as appropriate to the LTC).

Starting SCS early on in the disease course (Boon et al., 2020; Revert et al., 2018; Stapleton, 2001) negated having to change established behaviours and reverse poor metabolic control. Significant challenges were encountered where there was a wide variation in how long study participants had been diagnosed with their LTC or had been performing dietary self-care. For example, between one and 17 years since diagnosis for participants with T1DM (Coates et al., 2013), between one and 11.7 years since starting a gluten-free diet (Connan et al., 2019) and between 6 months and 9 years already counting CHOs (Spiegel et al., 2012). In the study by Christie et al. (2016), participants with the highest HbA1c (poorer metabolic control) were less likely to attend group education sessions; in addition, significantly more children (8–12 years) attended, compared with teenagers (13–16 years) (*n* = 62, 64% vs. *n* = 42, 44%). This finding was consistent across several studies, where children in the younger age groups were the more receptive and keener to learn (Bell, 2004; Culhane, 2013; Fishman et al., 2018; Owen et al., 2013).

### Keeping it going

Across the included studies, models of SCS were more successful when the intervention or exposure to SCS was of longer duration. For example, between six and 12 months (Boon et al., 2020; Cooper et al., 2018; Fiallo-Scharer et al., 2019; Owen et al., 2013) to over 3 years (Revert et al., 2018). Provision of ongoing input, with regular reiteration of topics to reinforce knowledge and skills (Owen et al., 2013), correct misconceptions, misinformation or fill gaps (Culhane, 2013), together with further top-ups, enabled tailoring of SCS to meet specific and changing needs of children over time. This was not possible where SCS was delivered, for example, as an intensive 5-day block (Nabors et al., 2014; Price et al., 2016; Singh et al., 2000) or as a very brief intervention, for example, two six-hour group sessions (Cottrell et al., 1996) or viewing a CD-ROM for approximately 30 min (Davis et al., 2004).

Whilst provision of regular ongoing SCS may enable behaviours leading to the formation of self-care habits and routines, this was poorly addressed in the included studies.

### Being flexible

Models of SCS across the included studies employed various modes of delivery to accommodate differing needs and preferences. Study participants engaged well when SCS utilised a range of interactive (rather than passive) learning (Bell, 2004; Boon et al., 2020; Connan et al., 2019; Owen et al., 2013; Price et al., 2016; Spiegel et al., 2012; Stapleton, 2001). For example, adolescents with T1DM practised CHO counting in practical cookery sessions (Price et al., 2016) and children with CF learnt the fat content of foods though doing hands-on labelling activities and matching pair games (Owen et al., 2013).

SCS interventions that were integrated into routine clinic visits (Bell, 2004; Culhane, 2013; Fiallo-Scharer et al., 2019; Revert et al., 2018), either in a group-based format or on an individual basis, were not without challenges, chiefly due to time constraints in busy clinics. However, further challenges were encountered when SCS was delivered as an optional extra. For example, in groups in the clinic setting but independent to regular outpatient clinic (Christie et al., 2016; Coates et al., 2013), HCPs were trying to organise and deliver sessions in addition to their usual workload, often following little or no training; children and families also had competing demands, for example, school and work commitments. In a 10-week home-based programme, though carers enjoyed helping their child learn and learning themselves, some carers reported being too busy to easily fit in daily recording and weekly paper-based exercises with their child (Stapleton, 2001). Further to this, given the choice of completing the ADNAT app at home or in clinic, the majority of adolescents chose clinic (Cooper et al., 2018) (though this relied on having access to Wi-Fi in clinics) and for individual sessions with a dietitian, incorporating these as part of outpatient clinic visits was preferred over separate home visits (Owen et al., 2013).

Across the included studies, it was clear that integrating SCS into routine care required organisational commitment, with prioritisation and active support of HCPs at a service level (Christie et al., 2016; Cooper et al., 2018; Revert et al., 2018).

### Choosing appropriate outcomes

Evaluating success of the models of SCS relied on the choice of appropriate outcomes. Many of the included studies chose outcomes commonly used in clinical practice. For example, HbA1c as a measure of glycaemic control (Christie et al., 2016; Coates et al., 2013; Cooper et al., 2018; Fiallo-Scharer et al., 2019; Frøisland and Årsand, 2015; Price et al., 2016; Spiegel et al., 2012) and weight or BMI z-score as a measure of nutritional status (Boon et al., 2020; Coates et al., 2013; Cottrell et al., 1996; Owen et al., 2013; Revert et al., 2018; Stark et al., 2009). However, such outcomes may not be sensitive enough to detect clinically meaningful change or sustained behaviour change over the short duration of SCS interventions observed in the majority of included studies. Perhaps surprisingly (for studies related to SCS of diet and the gut), few included patient-reported outcomes, such as control of symptoms or quality of life (the exceptions being Christie et al., 2016; Cottrell et al., 1996; Fiallo-Scharer et al., 2019; Price et al., 2016), and only one study focused on GI-related quality of life (Boon et al., 2020).

The limited choice of outcomes in the included studies may, in part, reflect how the majority of SCS interventions lacked a theoretical basis to their development. Only four of the 19 intervention studies reported using an underlying theory or model of behaviour change (Cooper et al., 2018; Price et al., 2016; Singh et al., 2000; Stapleton, 2001). However, more encouragingly, eight of the 19 intervention studies (Boon et al., 2020; Christie et al., 2016; Coates et al., 2013; Connan et al., 2019; Cooper et al., 2018; Fiallo-Scharer et al., 2019; Price et al., 2016; Stapleton, 2001) reported involvement of patients and families in their development.

## Discussion

The aim of this integrative review was to identify, critically appraise and synthesise evidence from primary studies on SCS of diet and the gut in school-age children with LTCs. Synthesis of the 25 eligible studies identified six themes that collectively contribute new understanding of what constitutes SCS of diet and the gut, together with key requisites for models of SCS in the context of delivery and uptake in routine care.

SCS of diet and the gut throughout the school-age years was found to be complex and dynamic, yet on a continuum as the child grows. It included supporting stepwise development and application of a specific knowledge and skill set. This may be facilitated by the use of age/developmental stage competency checklists (Bell, 2004; Culhane, 2013; Fishman et al., 2018), such as those currently used in UK practice with children and adolescents with T1DM (Thornton et al., 2016) and in the USA with children and adolescents with CF (CF R.I.S.E, 2016). Particular emphasis is needed on numeracy skills, with a means of assessment and tailored support as appropriate (Moosa and Segal, 2011; Mulvaney et al., 2013). Visual resources may also be required. However, as images can be interpreted in many ways, involving children in the design and selection of images is essential to ensure images are meaningful to them and evoke positive emotional responses (Houts et al., 2006). Further studies are also needed combining visual resources with hands-on practical experience, as this may have more impact than visualisation alone (Evans et al., 2009).

SCS of diet and the gut also included providing practical help with incorporating the demands of self-care into everyday life. This encompassed attention to the fine detail, to enable both proactive and responsive tailoring of support. Further work is needed to identify how this collaborative approach can be implemented, for example, regarding expectations and roles of children, families and HCPs (Smith and Kendal, 2018). The included studies did not address the development of routines and habits in sustaining daily self-care. However, when treatment burden is high, routine is key to motivation (Calthorpe et al., 2020). More research is needed on how best to support habit formation throughout childhood and whether self-care behaviours established during childhood can be maintained despite challenges (such as lack of time and competing demands) during adolescence and through to adulthood (Hoo et al., 2019; Lally and Gardner, 2013).

In the included studies, models of SCS were more successful when started early on in the disease course and were ongoing, to allow tailoring to changing needs and priorities over time. In addition, to remain relevant, models need to adapt to advances in treatments and technologies. For example, in the new era of modulator therapies in CF, increased fat absorption and decreased gut inflammation may contribute to weight gain (Stallings et al., 2018), prompting greater emphasis on diet quality (Sutherland et al., 2018) and a more individualised approach to diet and PERT (McDonald et al., 2020).

Implementation of SCS was more successful when SCS interventions were embedded within routine care, rather than being an optional extra. However, there was no one size fits all; flexibility is needed in terms of what SCS interventions and activities can be accessed (such as interactive e-learning, mobile apps (Day, 2020), hands-on activities and learning through fun play sessions (La Banca et al., 2020)) to meet individual preferences and target specific needs at any one time. This complex picture was further compounded by the need for a whole system approach, where there is strong leadership and organisational support for HCPs to implement SCS in routine care, as reported previously (Taylor et al., 2014). To enable this, further studies are needed to identify training and supervision needs of HCPs and how system constraints such as limited consultation times and workload pressures can be adjusted (Eaton et al., 2015).

Outcomes of the included studies reflected more of a focus on self-management than self-care, that is, taking care of one’s own condition rather than taking care of oneself (Pelicand et al., 2013). Further work is needed to develop models of SCS that more accurately reflect the broad range of activities involved in self-care, identify outcomes that capture that breadth (Feillet et al., 2010) as well as the shorter-, medium- and longer-term effects of SCS. To facilitate this, models of SCS need to be theoretically informed (Kirk et al., 2013) and co-developed with patients and their families, as highlighted previously (Bee et al., 2018; Kirk et al., 2013). Furthermore, outcomes of SCS of diet and the gut of most value to patients and their families need to be explored (De Wit et al., 2020; Kirk et al., 2013; Ye et al., 2017).

### Implications for practice

The findings of this review suggest several implications for practice. Enabling children to gradually take an active role from early on in their care, requires a more child-centred focus (Coyne et al., 2016). HCPs need to support children to have time, space and repeated opportunities to apply their developing knowledge and skills, alongside helping to address factors that may affect their motivation to self-care. A greater emphasis on the health promotion aspect of self-care, adopting a more proactive, less reactive approach, will also require more time and potentially additional training and support for HCPs. Investment in SCS for the long term within organisations will therefore need the support of policy makers.

### Strengths and limitations of this review

In this review, varied data sources were used to identify primary research of all study designs, in both published and grey literature. Whilst this allowed exploration of multiple aspects of SCS of diet and the gut, analysis and synthesis of data from such a diverse range of studies are complex and can introduce bias and inaccuracy (Whittemore and Knafl, 2005). To combat this, a systematic and rigorous approach was adopted, with involvement of multiple authors at each stage. Most studies were conducted in high-income countries; however, the results may also be relevant in low- and middle-income countries. The methodological quality of some of the included studies (as presented in Supplementary Material 4) is a limitation, as is the inclusion of only English language studies, as some relevant non-English studies may have been omitted.

## Conclusion

Findings from this review suggest what SCS of diet and the gut for school-age children with LTCs consists of and indicate key requirements for models of SCS to work in the context of routine care. As such, the findings form a foundation for further work. In particular, more research is needed directly with children, their families and HCPs to advance understanding of their needs and preferences for SCS and inform development of a theory- and evidence-based model for SCS of diet and the gut.

## Supplemental Material

sj-pdf-1-chc-10.1177_13674935211029124 – Supplemental Material for Self-care support of diet and the gut in the routine care of school-age children with long-term conditions: An integrative review Click here for additional data file. Supplemental Material, sj-pdf-1-chc-10.1177_13674935211029124 for Self-care support of diet and the gut in the routine care of school-age children with long-term conditions: An integrative review by Laurie Cave, Linda J Milnes and Gretl A McHugh in Journal of Child Health Care

sj-pdf-2-chc-10.1177_13674935211029124 – Supplemental Material for Self-care support of diet and the gut in the routine care of school-age children with long-term conditions: An integrative review Click here for additional data file. Supplemental Material, sj-pdf-2-chc-10.1177_13674935211029124 for Self-care support of diet and the gut in the routine care of school-age children with long-term conditions: An integrative review by Laurie Cave, Linda J Milnes and Gretl A McHugh in Journal of Child Health Care

sj-pdf-3-chc-10.1177_13674935211029124 – Supplemental Material for Self-care support of diet and the gut in the routine care of school-age children with long-term conditions: An integrative review Click here for additional data file. Supplemental Material, sj-pdf-3-chc-10.1177_13674935211029124 for Self-care support of diet and the gut in the routine care of school-age children with long-term conditions: An integrative review by Laurie Cave, Linda J Milnes and Gretl A McHugh in Journal of Child Health Care

sj-pdf-4-chc-10.1177_13674935211029124 – Supplemental Material for Self-care support of diet and the gut in the routine care of school-age children with long-term conditions: An integrative review Click here for additional data file. Supplemental Material, sj-pdf-4-chc-10.1177_13674935211029124 for Self-care support of diet and the gut in the routine care of school-age children with long-term conditions: An integrative review by Laurie Cave, Linda J Milnes and Gretl A McHugh in Journal of Child Health Care
