# Supplementary material for: Self-care support of diet and the gut in the routine care of school-age children with long-term conditions: An integrative review
Source: J Child Health Care. 2021 Jun 30;26(4):668–82. doi: 10.1177/13674935211029124 (PMC9667094; doi:10.1177/13674935211029124)
Supplement: sj-pdf-1-chc-10.1177_13674935211029124 – Supplemental Material for Self-care support of diet and the gut in the routine care of school-age children with long-term conditions: An integrative review [file sj-pdf-1-chc-10.1177_13674935211029124.pdf]

S1. Inclusion and exclusion criteria for two-stage screening (stage 1, titles and abstracts; stage 2, full-text eligibility)

Criteria	Inclusion	Exclusion
**Sample: Participants and conditions**	Focus on<br>• Children of compulsory school age (4-16 years old), or<br>• Child-parent/carer dyads	• Mean age of children reported as under 4 years of age or over 16 years of age<br>• Focus on parents/carers only
	Children with<br>• Any physical LTC with a diet or GI-related component of care, or<br>• Any of the following: CF, T1DM, Coeliac Disease, Phenylketonuria or Inflammatory Bowel Disease (selected as they each require lifelong dietary treatment/ modification)	• Children with non-physical LTCs e.g., mental health conditions<br>• No specific diet/GI component
**Phenomena of Interest**	• Engagement or active involvement of children in some aspect of self-care of diet and/or the gut, having received some type of SCS (no restrictions regarding the format or mode of delivery of SCS)<br>• Enablers for and barriers to, delivery and uptake of SCS	• Focus on perspectives/ perception of self-care only<br>• Children's involvement in self-care of their parents or siblings LTC<br>• Transition from child to adult health services<br>• (except where a study relates to supporting increased independence for self-care pre-transition)
**Design**	• No restriction - qualitative, mixed methods and quantitative studies of all designs	
**Research type**	• Published literature and grey literature sources such as conference proceedings, dissertations and theses	• Editorials, commentaries/ opinion papers and protocols

CF: cystic fibrosis; GI: gastrointestinal; LTC: long-term condition; SCS: self-care support; T1DM: type 1 diabetes
