# Supplementary material for: Self-care support of diet and the gut in the routine care of school-age children with long-term conditions: An integrative review
Source: J Child Health Care. 2021 Jun 30;26(4):668–82. doi: 10.1177/13674935211029124 (PMC9667094; doi:10.1177/13674935211029124)
Supplement: sj-pdf-2-chc-10.1177_13674935211029124 – Supplemental Material for Self-care support of diet and the gut in the routine care of school-age children with long-term conditions: An integrative review [file sj-pdf-2-chc-10.1177_13674935211029124.pdf]

S2. Example of the search strategy used in Medline

**No.**	**Searches**
1	Child/ or child*.mp.
2	(school age child* or school-age child* or school child*).mp.
3	preadolescen*.mp.
4	Adolescent/ or adolescen*.mp.
5	(teen* or teenager*).mp.
6	youth.mp.
7	(juvenile* or p*ediatric).mp.
8	(young adj (person or people)).mp.
9	((child- adj (parent* or carer* or caregiver*)) or family).mp.
10	Chronic Disease/
11	((chronic* or persist* or ongoing) adj (disease* or condition* or illness*)).mp.
12	((longterm or long term or long-term) adj (disease* or condition* or ill*)).mp.
13	((gastrointestinal or digestive or GI related) adj (disease* or condition* or illness*)).mp.
14	Cystic Fibrosis/ or cystic fibrosis.mp.
15	Diabetes Mellitus, Type 1/
16	Inflammatory Bowel Disease*/
17	Celiac Disease/ or c*eliac disease.mp.
18	Phenylketonuria*/
19	(self care or self-care).mp.
20	Self Care/
21	(self management or self-management).mp.
22	(self help or self-help).mp.
23	(self efficacy or self-efficacy).mp.
24	(collaborati* adj (care or manag*)).mp.
25	((shared or support*) adj (care or manag*)).mp.
26	Diet/ or diet*.mp.
27	nutrition*.mp.
28	gut.mp.
29	((dietary self care or dietary self-care or dietary management) adj support).mp.
30	(coach* or training or program*).mp.
31	(education or skills or information or behavio*r).mp.
32	(engagement or involvement or empowerment or participation).mp
33	(autonom* or responsibility or adapta* or independen*).mp.
34	peer support.mp.
35	(decision* adj (shar* or support* or aid* or making)).mp.
36	(goal set* or problem solving).mp.
37	(plan or action plan* or checklist).mp.
38	1 or 2 or 3 or 4 or 5 or 6 or 7 or 8 or 9
39	10 or 11 or 12 or 13 or 14 or 15 or 16 or 17 or 18
40	19 or 20 or 21 or 22 or 23 or 24 or 25
41	26 or 27 or 28 or 29
42	30 or 31 or 32 or 33 or 34 or 35 or 36 or 37
43	38 and 39 and 40 and 41 and 42
44	limit 43 to (english language and humans and yr="1990 -Current")
