# Supplementary material for: Self-care support of diet and the gut in the routine care of school-age children with long-term conditions: An integrative review
Source: J Child Health Care. 2021 Jun 30;26(4):668–82. doi: 10.1177/13674935211029124 (PMC9667094; doi:10.1177/13674935211029124)
Supplement: sj-pdf-3-chc-10.1177_13674935211029124 – Supplemental Material for Self-care support of diet and the gut in the routine care of school-age children with long-term conditions: An integrative review [file sj-pdf-3-chc-10.1177_13674935211029124.pdf]

S3. Characteristics of the included studies

(Author, Year) Country	Research aim	Participants (LTC, age, sample size)	Design and methods	SCS Intervention/ exposure (including setting)	Key findings
(Austin et al., 2013) Canada	Test a model of motivational factors for dietary SC over a 24/12 interval	Adolescents with T1DM aged 11-17 yrs n=289 (n=237 at 24/12 F/U)	Quant descriptive; questionnaires at baseline and 24/12	Routine support/ recommended dietary SC programme at 2 DM centres	Autonomy support from HCPs positively predicted autonomous self-regulation and SE, which in turn predicted better dietary SC over 24/12 interval. SE perception was positively related to subsequent parental autonomy support.
(Austin et al., 2011) Canada	Examine the relationships between metabolic control, dietary SC and motivation	Adolescents with T1DM aged 11-17 yrs n=289	Quant descriptive; questionnaires at baseline	Routine support/ recommended dietary SC programme at 2 DM centres	Overall metabolic control was suboptimal. Better metabolic control was associated with better dietary SC. Interventions should aim to encourage parents to be less controlling. No gender differences.
(Bell, 2004) UK	Development and evaluation of a nutrition and enzyme education programme for children with CF	Children with CF aged 5-11 yrs and parents	Quant descriptive; questionnaires at baseline and post development	Pen and paper-based 13 module structured education programme; used in O/P clinic and during admissions at n=13 CF centres	Modules used most with 5-10-year-old children - key time as most receptive. When re-evaluated in 2011, UK Dietitians were using individual modules as needed - the 'programme' was never used as it was envisaged.

(Author, Year) Country	Research aim	Participants (LTC, age, sample size)	Design and methods	SCS Intervention/ exposure (including setting)	Key findings
(Boon et al., 2020) Belgium	Assess effect of a mobile app (MyCyFAPP) for PERT adjustment (+ nutrition education tools and resources) on GI-related QoL in children with CF	Children with CF aged 2-18 yrs n=171	MM; quant non-randomised: 6/12 prospective clinical trial at 6 CF centres in 5 European countries incorporating QoL questionnaires; qual: interviews n=41. No control group.	Use of mobile app for patients and parents (and a professional web tool for HCPs to evaluate patient's data and give feedback) over 6/12	GI-related QoL significantly improved (symptoms reduced), with similar results obtained from patients and parents. Overall median PERT dose did not change. For pts and parents: enzyme dose calculations were used most. For HCPs (particularly dietitians): more precise info obtained prior to consultations allowing for more personalised advice but quality of recorded data decreased over time. The app enables rapid increase in knowledge to implement treatment; regular app usage over a long period is not needed.
(Christie et al., 2016) UK	Evaluate effectiveness of a clinic-based structured educational DM Ix (CASCADE) for children and adolescents with poorly controlled T1DM	Children and adolescents with T1DM aged 8-16 yrs Ix n=159, control n=168; parent/carer Ix n=156, control n=169	Quant RCT comparing Ix to usual care at 28 cluster-randomised paediatric DM clinics; 12 and 24/12 F/U post Ix	Manual-based 4 module structured education programme; groups of 3-4 families to attend 1 module/ month (2hrs) in clinic over 4/12. Most groups offered during standard clinic times	Ix did not improve HbA1c at 12 or 24 months; no significant change in other outcomes. Attendance insufficient to demonstrate an Ix effect: only 55/180 (30%) received the full 4 modules. Significant variation in 'usual care' (controls) across sites. Ix may have been more effective if aimed at children with lower HbA1c and earlier in their DM history.

(Author, Year) Country	Research aim	Participants (LTC, age, sample size)	Design and methods	SCS Intervention/ exposure (including setting)	Key findings
(Coates et al., 2013) UK	Evaluate effectiveness of a structured DM education programme (CHOICE) on glycaemic control and dietary adherence for adolescents with T1DM	Adolescents with T1DM aged 13-19 yrs; Ix n=70, controls n=65	Quant RCT comparing Ix to usual care at 7 hospital sites, parallel design	3-hrly interactive group-based sessions over 4/52 focusing on insulin adjustment to liberate diet and lifestyle; text support at 2, 4 and 5/12; 24/12 F/U post Ix. Clinic setting but delivered independently to regular O/P clinic.	No significant difference in HbA1c between groups at 12/12 (n=57 Ix, 43 control) despite a much more liberal diet in the Ix group but at 24/12 (n=31 Ix, 28 control) HbA1c significantly higher in Ix group. No difference in BMI or hyper and hypoglycaemic episodes. Dietary adherence deteriorated by 12/12 post Ix and in both Ix and controls by 24/12. Single education Ix (of 12hrs) inadequate - need further support/ input following the 4/52 programme ('top up' over time).
(Connan et al., 2019) Canada	Assess usability of an interactive e-learning module about GFD for children with CD and T1DM and their caregivers	Children with concurrent CD and T1DM mean age 13.5 yrs n=18 and their caregivers n=15	MM; quant descriptive: pre and post knowledge questionnaires, then qual: observation of 60-min usability test of module with think-aloud + semi-structured interviews	Interactive e-learning module on GFD; DM and CD clinics at a single centre	Knowledge test scores increased significantly from pre to post module completion. Pts and caregivers reported module contained lots of useful info for those newly diagnosed with CD. Most participants envisioned module would be best suited for 8-9 yrs and older and a more game-centred or interactive module would be required for younger children.

(Author, Year) Country	Research aim	Participants (LTC, age, sample size)	Design and methods	SCS Intervention/ exposure (including setting)	Key findings
(Cooper et al., 2018) UK	Evaluate the feasibility of integrating an app (ADNAT App) into UK paediatric DM care	Adolescents with T1DM aged 12-18 yrs n=89 (44 completers and 45 non-completers	MM; quant non-randomised Ix study at 3 sites; completers (and submitters of ADNAT questionnaires) compared with non-completers (+ survey and focus groups with HCPs)	ADNAT App for 6/12; combines reflective questioning with needs Ax; 6 domains (eating is 1 domain). 3 DM centres in England; participants could choose to complete it at home and/or in clinic.	Large baseline differences in HbA1c and variable rates of change at 6/12; after adjusting for baseline HbA1c and site, completers had a lower post Ix mean HbA1c than non-completers at 6/12. Pt's HbA1c at 6/12 correlated reasonably well with their ADNAT scores.
(Cottrell et al., 1996) USA	Evaluate effectiveness of a self-management training programme for CF	Children with CF aged 8-18 yrs n=20 and their parents (10 children and 10 parents in Ix group and control group)	Quant RCT comparing Ix to usual care at baseline and 6-8/52 post Ix, parallel design; single site	Two 6hr group sessions at CF centre. Manual based. Timeframe between 6-hr sessions not stated. 2/52 self-monitoring and return of records pre and 6-8/52 post Ix.	No significant difference in change in wt, SM behaviours (children and parents), children's adherence or quality of well-being or parents' knowledge between groups at 6-8/52 F/U, though children's knowledge about CF and its management was greater in the Ix group suggesting the Ix could increase children's knowledge in the short-term.
(Culhane, 2013) USA	Development and evaluation of an in-clinic nutrition education tool for children with CF	Children with CF (all ages) n=205 and their parents	Quant descriptive; baseline and post Ix knowledge questionnaires at a single CF centre	Pre-test of nutrition knowledge followed by education based on identified knowledge deficits. Tailored to age of child. O/P clinic.	Some retention of nutrition knowledge seen at F/U, but annual reinforcement helpful in continuing to educate patients/families. Nutrition checklist continues to be used in practice at annual review. A brief evaluation of BMI trends was conducted 1 year after implementation and an increase in BMI percentiles was found. No further re-evaluation of its use or effectiveness to date.

(Author, Year) Country	Research aim	Participants (LTC, age, sample size)	Design and methods	SCS Intervention/ exposure (including setting)	Key findings
(Davis et al., 2004) USA	Evaluate effectiveness of the STARBRIGHT *fitting CF into your everyday life* CD-ROM	Children and adolescents with CF; treatment: mean age 13.6 yrs n=25 and wait-list controls: mean age 12.5 yrs n=22	Quant RCT; crossover design: treatment group received Ix, wait-list group served as controls both for time and maturation then received Ix. Pre and post assessment of CF-related knowledge and coping skills using questionnaire and audiotaped vignettes	View CD-ROM for approx. 30 minutes during an O/P clinic visit - 3 modules, 1 on eating	Knowledge improved and small effects found for changes in coping strategies immediately after viewing the CD-ROM in both treatment and wait-list groups. Ix very brief - no F/U so? how long children and adolescents were able to retain this info. Additional studies needed to address whether verbally generated strategies lead to behaviour change and whether the modest improvements are clinically meaningful.
(Fiallo-Scharer et al., 2019) USA	Evaluate impact of an Ix that tailored delivery of T1DM SM resources to families' specific SM barriers	Children with T1DM aged 8-16 yrs n=106 and controls n=108 and their parent(s)	Quant RCT comparing Ix to usual care at 2 sites, parallel design; 12/12 Ix and 12/12 F/U post Ix	PRISM (problem recognition in illness self-management) survey to identify 3 of 5 family SM barriers, received usual care + up to 4 x 75-min tailored SM resource group sessions pre or post routine DM clinic visits	82% of families attended at least half of the group sessions. No overall Ix effect on HbA1c or QoL found; for 13-16yr olds at 1 site, significant improvements in post-Ix HbA1c and mean QoL of parents of 8-12 yr olds found; Ix may benefit specific children, especially those with high baseline HbA1c. Improvements in HbA1c were largely due to resources addressing barriers to motivation to SM.

(Author, Year) Country	Research aim	Participants (LTC, age, sample size)	Design and methods	SCS Intervention/ exposure (including setting)	Key findings
(Fishman et al., 2018) USA	To create a patient-centred age-appropriate skill acquisition list specific to CD SM in children	Pts with CD mean age 12 yrs n=204 and parents n=155	Quant descriptive; expert group consensus to develop list of tasks specific to CD SM, from which parallel surveys developed for pts and parents re: age at which an 'average child with CD' would master each task	N/A; study included as an enabler of SCS O/P clinic at a single Children's Hospital	The earliest tasks were mastered by a median age of 8 yrs; pts and parents reported similar ages for skill mastery. Many tasks for diet-based SM are mastered at a far younger age compared with general timelines for starting the transition process.
(Frøisland and Årsand, 2015) Norway	Evaluate effect of a mobile-phone-based tool to visualise food intake with regard to empowerment, SE and self-treatment of T1DM	Adolescents with T1DM aged 13-19 yrs n=12	MM; quant non-randomised: pre-post 3/12 pilot of an Ix - 2 smartphone apps - DiaMob app and the Diabetes message system (DMS), consultation midway, then qual: semi-structured interview at end of 3/12	DiaMob app - 2 x 3-day recordings were mandatory - take photos of own foods to target evaluation of CHO and insulin dosage, invited to use DMS to send short messages to their providers; consultation midway; 2 O/P clinics	Both apps useful support for DM SM. DiaMob promoted visual understanding and more accurate estimation of CHO; adolescents reported improved knowledge and skills, attitudes and self-awareness. DMS - enabled them to take action and be in charge; liked text messages with simple practical advice. HbA1c improved in 7 of the 11 participants who completed the study.

(Author, Year) Country	Research aim	Participants (LTC, age, sample size)	Design and methods	SCS Intervention/ exposure (including setting)	Key findings
(Kyngäs et al., 1998) Finland	Explore adolescents' perceptions of how the actions of HCPs, family and friends help or hinder their compliance with T1DM SC	Adolescents with T1DM aged 13-17 yrs n=51	MM: qual (interviews, content analysis) + quant descriptive (questionnaires re: SC compliance with insulin treatment, diet, home monitoring, co-operation with HCPs)	Routine support	Good compliance with SC when: physicians and nurses actions were motivating (ask, listen and take notice of adolescent's opinion; plan SC together), parents motivating or accepting (show interest in them, accept them as they are, provide positive feedback, help solve problems associated with SC and fitting it into everyday life, do not try to take too much control), friends providing silent support and acceptance.
(Nabors et al., 2014) USA	Evaluate children's learning and goal attainment related to change in their SM skills during a T1DM camp	Children with T1DM aged 8-16 yrs n=131 (in 2011) and n=68 (in 2012)	Quant descriptive; cross-sectional surveys: parent report before camp of child goals + child report at end of camp of what they learned	1/52 DM camp in 2011 and 2012; in 2011, parents reported what their child needed to learn + children independently selected goals, in 2012 parent and child shared goal-setting for SM was added.	Children learnt about recognising and managing hypo and hyperglycaemia, eating healthily, improved ability to independently count CHOs etc. Boys reported learning more about managing T1DM, whereas girls reported the value of opportunities to express feelings about coping with and managing DM. There was not a strong match between parent goals and children's learning, though joint goal-setting was more successful.

(Author, Year) Country	Research aim	Participants (LTC, age, sample size)	Design and methods	SCS Intervention/ exposure (including setting)	Key findings
(Owen et al., 2013) UK	Evaluate impact of a pilot intensive (joint physiotherapy and) dietetic education Ix on nutrition knowledge and nutritional status in children with CF	Children with moderate to severe CF aged 5-15 yrs n=15	Quant descriptive; baseline and post Ix knowledge questionnaires (+ patient satisfaction survey) at a single CF centre	Nutrition and PERT education; practical activities and 6 individualised teaching sessions of 30-60mins delivered 1-2/12 over 12/12 at home, on ward or in O/P clinic.	Nutrition quiz scores higher post Ix suggesting improvement in knowledge. The majority of children maintained their BMI z-scores within 0.5 of their baseline measurements (note exercise capacity increased). Younger children (5-10yrs) benefited from shorter sessions (20-30mins) and needed regular reiteration of topics to reinforce knowledge. Author suggests targeting 8-11yr olds as most receptive and incorporate sessions into structured O/P clinic visit.
(Price et al., 2016) UK	Determine the effectiveness of the Kids in Control of Food (KICk-OFF) structured intensive education course for T1DM	Adolescents with T1DM aged 11-16 yrs n=198 and controls n=194	Quant RCT with Ax at baseline & F/U at 6/12, 1yr and 2yrs post Ix; cluster-randomised	5-day group education focusing on SM skills for CHO counting and insulin adjustment; usual care and education for control group. 31 DM centres (17 Ix, 15 control), delivered in community setting.	Participation was associated with improved overall QoL within 6months. No difference in HbA1c between control and Ix groups. At 1 yr and 2yrs post Ix the control group had significantly higher scores for adherence to treatment and at 6/12 and 1 yr, significantly higher SE scores than the Ix group. Authors suggest improved and sustained glycaemic control may require educational interventions delivered from diagnosis as a fundamental component of care and not an 'optional extra'.
(Rankin et al., 2018a) UK	Explore barriers and facilitators for taking on DM SM tasks	Children with T1DM aged 9-12 yrs n=24	Qual; interviews; thematic	Routine support; 4 DM centres	Barriers to children taking on new SM responsibilities: over-reliance on parents, lacking maths skills to count CHO and determine insulin doses. Motivators/tipping points to taking on SM responsibilities: alleviate burden on parents, spend time with their friends, preparing to start secondary school.

(Author, Year) Country	Research aim	Participants (LTC, age, sample size)	Design and methods	SCS Intervention/ exposure (including setting)	Key findings
(Rankin et al., 2018b) UK	Identify how children can be better supported by friends and peers to undertake DM SM			DM-related support from friends and peers; 4 DM centres	Informing peers (classmates) about T1DM often resulted in unwanted attention; peers struggled to understand T1DM and could be insensitive and unsupportive. Children had mixed views and ambivalent views about receiving support from other children with T1DM. HCPs could consider ways to assist small friendship groups to undertake monitoring and prompting, practical help with SM tasks and normalising roles.
(Revert et al., 2018) France	Evaluate implementation of a therapeutic patient education programme to improve nutritional status of children with CF	Children with CF aged 2-12 yrs n=34 in 2011 and n=44 in 2014	Quant non-randomised; prospective longitudinal cohort at a single CF centre	3yr nutrition education programme integrated into routine practice alongside intensification of F/U (according to nutritional risk status of individual children)	Nutritional status (median BMI z-scores) improved and mean FEV1 (for children>5 yrs) showed no decline over the 3 yrs. Parents and children acquired skills and autonomy (Note: no detail re: skills). The relationship between professionals, pts and parents was strengthened. Programme integrated into routine practice since 2014 and extended to include 1 month -18 yr olds.
(Singh et al., 2000) USA	Evaluate effectiveness of an education Ix in a camp setting on knowledge, attitudes and beliefs about PKU diet	Adolescent girls with PKU mean age 13 yrs n=13 1st-year campers compared with n=11 in 2nd yr, n=8 yr 3 and n= 7 yr 4	Quant non-randomised; pre-post Ix questionnaires, 3/7 diet records and blood tests and at F/U at 4, 8 and 12/12	Diet and disease education at a 1/52 metabolic summer camp alongside recreational activities.<br>Study in yr 1 and 3 subsequent yrs of camp.	Short-term effects: significant improvement in metabolic control, knowledge of diet, fewer barriers to complying with PKU diet, less perceived isolation – however, these effects progressively returned to baseline levels over 1yr. The long-term decreased dietary compliance was associated with lack of support, feelings of peer rejection, and increased barriers due to lack of info and availability of Phe-restricted foods.

(Author, Year) Country	Research aim	Participants (LTC, age, sample size)	Design and methods	SCS Intervention/ exposure (including setting)	Key findings
(Sparapani et al., 2017) Brazil	Identify children's learning needs about T1DM, their self-care tasks and their video game preferences	Children with T1DM aged 7-12 yrs n=19	Qual; focus groups (7-9 yrs and 10-12 yrs); content analysis	Routine DM education at 1 DM centre; Ix development (video game)	Learning needs: dealing with emotions e.g. around food intake, motivation to eat healthily and be physically active, knowledge of food groups; lack of practical skills to effectively self-care e.g. CHO count, lack of awareness regarding influence of foods on glycaemic control and the function of insulin. Video preferences: to be able to see what happens inside their bodies as a result of T1DM, to learn about what they can and cannot eat and how to easily CHO count.
(Spiegel et al., 2012) USA	Determine whether a nutrition education Ix improves CHO counting accuracy and glycaemic control in adolescents with T1DM	Adolescents with T1DM aged 12-18 yrs n=66 who inaccurately count CHOs (n=33 Ix, n=33 control group)	Quant RCT comparing Ix to routine care at baseline and 3-4/12 at a single site	Interactive 90-minute nutrition education class with a dietitian/ certified DM educator (planned curriculum targeting adolescent's problem areas; completion of 2 x 3-day food records with tel feedback from the dietitian.	At baseline, CHOs were under or overestimated. There was no significant difference between the Ix and control groups in CHO counting or HbA1c at 3/12. No association found between duration of CHO counting and accuracy therefore regular re-education needed. Adolescents who collaborated more with their parents had lower HbA1c, therefore continued parental involvement in their DM care needs to be encouraged. More intensive education may be required.

(Author, Year) Country	Research aim	Participants (LTC, age, sample size)	Design and methods	SCS Intervention/ exposure (including setting)	Key findings
(Stapleton, 2001) Australia	Development, implementation and evaluation of a nutrition and PERT education and behaviour change programme 'Go and Grow with CF'	Children with CF aged 6-11 yrs n=41 (21 in Ix gp, 20 in control gp) and caregivers of 2-11 yr olds n= 54 (27 in Ix gp, 27 in control gp)	Quant RCT comparing Ix to routine care at baseline, end of Ix and 12/12 post Ix, parallel design; single site	10/52 home-based pen and paper course (children and their caregivers completed weekly exercises each lasting approx. 60 minutes) with introductory and concluding group workshops at hospital and monthly tel calls from dietitian.	Significant improvement in children's knowledge (nutrition and enzymes) at end of Ix, but not at 12/12 F/U. No statistically significant improvements in dietary intake or nutritional status. The apparent lack of a long-term effect of a single exposure to the programme on knowledge suggests that regular, ongoing education and counselling is required by families to reinforce aspects related to the child's stage of development and disease status. Author suggested a preventative approach to nutrition in CF in the form of several behavioural-based programmes throughout the child's life may be advantageous.
(Stark et al., 2009) USA	Evaluate efficacy of a behavioural plus nutrition education Ix, Be In CHARGE, in children with CF	Children with CF aged 4-12 yrs n=67 and their parent(s)	Quant RCT comparing behavioural plus nutrition education intervention (B+NE) with a nutrition education Ix (NE) at 5 sites, with 5 F/U Ax's up to 24/12 post Ix	7 group sessions over 9/52. Manual-based. Parents and children seen in simultaneous but separate groups. NE component same in NE and B+NE groups. 5 CF centres.	B+NE Ix was more effective than NE at increasing dietary intake and wt over a 9/52 period, however across the 24/12 F/U, both achieved similar outcomes. Authors suggest the dietary info in the NE Ix was very behavioural. [Currently being trialled as a 10/52 web-based Ix (instead of face-to-face) for children with CF aged 3-10 yrs (estimated completion date of feasibility trial Dec 2020)]

(Author, Year) Country	Research aim	Participants (LTC, age, sample size)	Design and methods	SCS Intervention/ exposure (including setting)	Key findings
(Witalis et al., 2017) Poland	Assess patients' and their parents' knowledge and attitudes towards compliance with the PKU diet	Patients with PKU aged 10-19 yrs n=173 (n=140 aged 10-16yrs) and their parents n=110 (+ PKU patients >20 yrs n=45)	Quant descriptive; questionnaires for patients and parents	Routine nutrition education (principles of dietary therapy including individual intakes of Phe and protein, menu planning and Phe, protein and calorie content of foods); 9 metabolic centres	Only 45% of patients knew daily Phe intake recommendations and 27% knew Phe content in the selected foods. Knowledge increased with the child's age, but knowledge was not associated with improved dietary compliance. Consistent, long-term family and individual therapy is required, with practical support for families to promote children's independence in meal selection and positive acceptance and motivation to follow PKU diet.

ADNAT: Adolescent Diabetes Needs Assessment Tool; Ax(s): assessment(s); BMI: body mass index; CASCADE: Child and Adolescent Structured Competencies Approach to Diabetes Education; CD: coeliac disease; CHO(s): carbohydrate(s); CHOICE: Carbohydrate, Insulin, Collaborative Education; CF: cystic fibrosis; DM: diabetes; F/U: follow up; GI: gastrointestinal; GFD: gluten-free diet; HCP(s): health care professional(s); Ix: intervention; MM: mixed-methods; O/P: outpatient; pt(s): patient(s); PERT: pancreatic enzyme replacement therapy; Phe: phenylalanine; PKU: phenylketonuria; QoL: quality of life; Qual: qualitative; Quant: quantitative; RCT: randomised controlled trial; SC: self-care; SE: self-efficacy; SM: self-management; T1DM: type 1 diabetes; wt: weight.
