# Supplementary material for: Self-care support of diet and the gut in the routine care of school-age children with long-term conditions: An integrative review
Source: J Child Health Care. 2021 Jun 30;26(4):668–82. doi: 10.1177/13674935211029124 (PMC9667094; doi:10.1177/13674935211029124)
Supplement: sj-pdf-4-chc-10.1177_13674935211029124 – Supplemental Material for Self-care support of diet and the gut in the routine care of school-age children with long-term conditions: An integrative review [file sj-pdf-4-chc-10.1177_13674935211029124.pdf]

S4. Findings of the quality appraisal using MMAT.

**Author**	**Screening Questions**		**1. Qualitative Studies**					**2. Randomized Controlled Trials**					**3. Non-randomized studies**					**4. Quantitative Descriptive Studies**					**5. Mixed Methods Studies**				
	S1	S2	1.1	1.2	1.3	1.4	1.5	2.1	2.2	2.3	2.4	2.5	3.1	3.2	3.3	3.4	3.5	4.1	4.2	4.3	4.4	4.5	5.1	5.2	5.3	5.4	5.5
Austin et al., 2013	Y	Y																Y	?	Y	Y	Y					
Austin et al., 2011	Y	Y																Y	Y	Y	?	Y					
Bell, 2004	N	N																									
Boon et al., 2020	Y	Y	Y	Y	Y	Y	Y						?	Y	Y	?	Y						Y	Y	Y	?	Y
Christie et al., 2016	Y	Y						?	Y	Y	Y	N															
Coates et al., 2013	Y	Y						?	Y	N	?	Y															
Connan et al., 2019	Y	Y	Y	Y	Y	Y	Y											Y	?	Y	?	Y	?	Y	Y	Y	Y
Cooper et al., 2018	Y	Y	Y	Y	Y	Y	Y						?	Y	Y	?	?						?	Y	Y	Y	Y
Cottrell et al., 1996	Y	Y						?	N	?	?	Y															

Screening questions and questions for each study design are listed on pages 4 and 5; Y=yes, N=no, ?=can’t tell

Author	Screening Questions		1. Qualitative Studies					2. Randomized Controlled Trials					3. Non-randomized studies					4. Quantitative Descriptive Studies					5. Mixed Methods Studies				
	S1	S2	1.1	1.2	1.3	1.4	1.5	2.1	2.2	2.3	2.4	2.5	3.1	3.2	3.3	3.4	3.5	4.1	4.2	4.3	4.4	4.5	5.1	5.2	5.3	5.4	5.5
Culhane, 2013	?Y	?Y																?Y	?Y	?Y	?Y	NA					
Davis et al., 2004	Y	Y						?	Y	Y	?	Y															
Fiallo-Scharer et al., 2019	Y	Y						?	?	Y	?	?															
Fishman et al., 2018	Y	Y																Y	Y	Y	?	Y					
Frøisland and Årsand, 2015	Y	Y	Y	Y	Y	Y	Y						?Y	?	?	N	?						?	Y	Y	?	?
Kyngäs et al., 1998	Y	Y	Y	Y	Y	Y	Y											?	?	?	?	?	?	?	?	?	?
Nabors et al., 2014	Y	Y																?	?	?	N	?					
Owen et al., 2013	Y	Y																Y	Y	?	Y	Y					
Price et al., 2016	Y	Y						Y	Y	Y	Y	Y															

Screening questions and questions for each study design are listed on pages 4 and 5; Y=yes, N=no, ?=can't tell

**Author**	**Screening Questions**		**1. Qualitative Studies**					**2. Randomized Controlled Trials**					**3. Non-randomized studies**					**4. Quantitative Descriptive Studies**					**5. Mixed Methods Studies**				
	S1	S2	1.1	1.2	1.3	1.4	1.5	2.1	2.2	2.3	2.4	2.5	3.1	3.2	3.3	3.4	3.5	4.1	4.2	4.3	4.4	4.5	5.1	5.2	5.3	5.4	5.5
Rankin et al., 2018a	Y	Y	Y	Y	Y	Y	Y																				
Rankin et al., 2018b	Y	Y	Y	Y	Y	Y	Y																				
Revert et al., 2018	Y	Y											Y	Y	Y	?	Y										
Singh et al., 2000	Y	Y											?	Y	?Y	Y	Y										
Sparapani et al., 2017	Y	Y	Y	Y	Y	Y	Y																				
Spiegel et al., 2012	Y	Y						?	Y	?	N	?															
Stapleton, 2001	Y	Y						?	Y	?	?	Y															
Stark et al., 2009	Y	Y						N	Y	?Y	N	?															
Witalis et al., 2017	Y	Y																Y	?Y	?	?	Y					

Screening questions and questions for each study design are listed on pages 4 and 5; Y=yes, N=no, ?=can't tell

SCREENING QUESTIONS

S1. Are there clear research questions?

S2. Do the collected data allow to address the research questions?

1.QUALITATIVE STUDIES

1.1. Is the qualitative approach appropriate to answer the research question?

1.2. Are the qualitative data collection methods adequate to address the research question?

1.3. Are the findings adequately derived from the data?

1.4. Is the interpretation of results sufficiently substantiated by data?

1.5. Is there coherence between qualitative data sources, collection, analysis and interpretation?

2. RANDOMIZED CONTROLLED TRIALS

2.1. Is randomization appropriately performed?

2.2. Are the groups comparable at baseline?

2.3. Are there complete outcome data?

2.4. Are outcome assessors blinded to the intervention provided?

2.5 Did the participants adhere to the assigned intervention?

3. NON-RANDOMIZED STUDIES

3.1. Are the participants representative of the target population?

3.2. Are measurements appropriate regarding both the outcome and intervention (or exposure)?

3.3. Are there complete outcome data?

3.4. Are the confounders accounted for in the design and analysis?

3.5. During the study period, is the intervention administered (or exposure occurred) as intended?

4. QUANTITATIVE DESCRIPTIVE STUDIES

4.1. Is the sampling strategy relevant to address the research question?

4.2. Is the sample representative of the target population?

4.3. Are the measurements appropriate?

4.4. Is the risk of nonresponse bias low?

4.5. Is the statistical analysis appropriate to answer the research question?

5. MIXED METHODS STUDIES

5.1. Is there an adequate rationale for using a mixed methods design to address the research question?

5.2. Are the different components of the study effectively integrated to answer the research question?

5.3. Are the outputs of the integration of qualitative and quantitative components adequately interpreted?

5.4. Are divergences and inconsistencies between quantitative and qualitative results adequately addressed?

5.5. Do the different components of the study adhere to the quality criteria of each tradition of the methods involved?
